# Multi-center QA of ultrahigh-field systems

**DOI:** 10.1007/s10334-025-01232-8

**Published:** 2025-03-24

**Authors:** Oliver Kraff, Markus W. May

**Affiliations:** 1 Erwin L. Hahn Institute for MR Imaging, University of Duisburg-Essen, Kokereiallee 7, 45141 Essen, Germany; 2 High-Field and Hybrid MR Imaging, University Hospital Essen, Essen, Germany

**Keywords:** Ultra-high field (UHF) MRI, 7 Tesla (7 T), Quality assurance (QA), Multi-center

## Abstract

Over the past two decades, ultra-high field (UHF) magnetic resonance imaging (MRI) has evolved from pure investigational devices to now systems with CE and FDA clearance for clinical use. UHF MRI offers enhanced diagnostic value, especially in brain and musculoskeletal imaging, aiding in the differential diagnosis of conditions like multiple sclerosis and epilepsy. However, to fully harness the potential of UHF, multi-center studies and quality assurance (QA) protocols are critical for ensuring reproducibility across different systems and sites. This becomes even more vital as the UHF community comprises three generations of magnet design, and many UHF sites are currently upgrading to the latest system architecture. Hence, this review presents multi-center QA measurements that have been performed at UHF, in particular from larger consortia through their “travelling heads” studies. Despite the technical variability between different vendors and system generations, these studies have shown a high level of reproducibility in structural and quantitative imaging. Furthermore, the review highlights the ongoing challenges in QA, such as transmitter performance drift and the need for a standard reliable multi-tissue phantom for RF coil calibration, which are crucial for advancing UHF MRI in both clinical and research applications.

## Introduction

Over the past two decades, magnetic resonance imaging (MRI) at ultra-high magnetic fields (UHF) has emerged from an investigational device installed at a few sites to more than 100 7 T systems in 2023 [1]. The number of installations worldwide is steadily increasing since the first system received clearance for clinical use in 2017 for brain and knee imaging [2]. As of today, two commercial vendors are offering clinical 7 T MR systems for sale [2, 3]. New installations are no longer exclusively found at universities with physics and engineering departments, but also at pure medical sites. Among other applications, 7 T MRI proved to be of added diagnostic value for the differential diagnosis between multiple sclerosis (MS) and other neuroinflammatory diseases [4], for identifying structural brain lesions in patients with drug-resistant focal onset epilepsy [5], as well as in assessing cartilage repair tissue in musculoskeletal questions [6]. To take the full advantage of promising UHF MR biomarkers for disease prognosis and treatment monitoring, multi-center studies are needed. In addition to qualitative imaging, for example by visualizing iron rim lesions to mark chronic active white matter MS lesions in a patient, quantitative mapping of biomarkers becomes more and more important in the arena of translational and precision medicine [7]. MRI can significantly increase the usefulness and informative value of quantitative imaging data, but requires careful standardization of techniques, and assessment of the repeatability and reproducibility of measurements across different manufacturers and software versions [8]. Furthermore, 7 T sites of the first and second system generation are currently upgrading to the latest 7 T platforms, necessitating quality assurance (QA) scans to track variations, both to verify the expectations of the investment, but also to determine its influence for studies that have not been completed before the upgrade [9].

Certainly, a vendor-provided QA monitoring is typically part of the maintenance contract between vendor and customer. However, especially during the first years of UHF MRI, established routines from 3 T needed to be optimized and adapted for 7 T. This included primarily adaptions to the increased mechanics of magnet and gradient coil interactions. After the introduction of higher channel counts for RF transmission and the advent of parallel transmission (pTx) techniques, supervision methods for phase differences, reflection, and coupling between elements, for example, needed additional attention in the vendor’s tune up and QA procedures. On the other hand, these developments paved the way for introducing two-port RF shimming at clinical 3 T systems. Likewise, 7 T was benefiting from new developments at lower field strengths. Since many research studies were comparing 7 T MR images with those obtained in the same subjects at 1.5 or 3 T, there was a mutual interest in optimizing protocols and procedures across all field strengths.

Nevertheless, research sites are always pushing the systems to the limit and the speed of research development is always exceeds the rate at which an MR system vendor can respond to new demands set by their customers. Furthermore, often perspectives on the matter substantially differed between MR system vendors, who were used to primarily deal with clinical customers and their usability of the system (i.e., rather short protocols), and pure research sites that were investigating much higher spatial resolutions in much longer scan acquisitions leading to an unprecedented demand on the system’s mechanics. Despite regular service visits, gradient failure was quite frequent and many first-generation gradient coils had to be replaced. This resulted in quite complex and long service visits delaying or even endangering elaborately planned research studies. Hence, it was and still is in the interest of the individual UHF MR site not only to implement additional QA monitoring, but also to perform cross-site comparisons.

The first 7 T multicenter study was performed in 2014 with the intention of harmonizing data acquisition and postprocessing of single voxel ^1^H MR spectroscopy in vivo at four different UHF sites [10]. Despite the challenges of a multi-vendor environment and the use of different RF head coils and transmission techniques, the authors were able to demonstrate high test–retest reproducibility of neurochemical concentrations between vendors. While there was a 25% difference in absolute values between MR systems of different MR vendors for macromolecular profiles, the absolute differences were 7% between the two Siemens systems in Essen, Germany and Minneapolis, MN, respectively, and 3% between the two Dutch Philips systems in Leiden and Utrecht [10], respectively. Over the past decade various consortia of 7 T sites have been formed within Europe [11]. Their aim was to facilitate and harmonize protocols, and to make UHF MR technology accessible to researchers and clinicians. Although the networks established QA procedures with dedicated phantoms, it is their travelling heads multi-center studies on structural and quantitative imaging that gained most interest. The two major networks, German Ultrahigh Field Imaging (GUFI) [12] and UK7T [13] from the United Kingdom, both scanned two subjects each at their UHF sites. Consequently, this in vivo data has been utilized for the purpose of QA and system performance comparison among the networks. This review describes the networks and the travelling heads studies in more detail, along with subsequently established other multi-center trials. After discussing challenges arising from non-standardized RF coils, this review will explore the need for dedicated phantoms and highlight key parameters requiring special attention at UHF.

### GUFI

The German Research Foundation (DFG) funded the GUFI consortium as a distributed national network from 2013 until 2021 [12]. Eleven German UHF sites who perform human MR imaging or spectroscopy at 7 and 9.4 T participated, along with several international partners who affiliated with the network over the time. Although all MR systems came from the same vendor (Siemens Healthineers AG, Germany), they featured different configurations in basic imaging components such as magnets, gradient and RF coils, or software versions. The network comprised all three generations of 7 T magnets, i.e., passively and actively shielded magnets of the first and second generation produced by Agilent Technologies, and the latest Terra magnet from Siemens, spanning up to 15 years of operation for the oldest systems. The main differences were identified in terms of two different sets of gradient coils (AS095 and SC72), two slightly different RF head coils (1-channel transmit and either 24-channel or 32-channel receive, both from Nova Medical, MA, USA), and two different software versions (VB17 and VE12).

To compare key system-related parameters and ensure that all systems were operating at peak performance, a common QA protocol was established [14]. In addition, a dedicated QA phantom was developed and rolled out to all sites of the GUFI network. The phantom had two compartments. A head-and-neck section was made from a PMMA tube, sealed at both ends with a half-shell and a plate, respectively, to fill the available space within the RF head coil. The container was filled with a gel of water, polyvinylpyrrolidone (PVP), agar and salt to emulate brain tissue (relative permittivity *ε*_r_ of 55, electrical conductivity *σ* of 0.6 S/m, T1/T2 of 700/60 ms). The second part of the phantom was designed to simulate the load of the human shoulders on the RF head coil and was therefore built as a rectangular container filled with a solution of water, PVP, and salt to mimic muscle tissue. High agreement was found between sites in SNR and flip angle measurements if the same type of RF coil was used. Inter-site differences were in the same range as the differences found in the longitudinal data analysis. Hardware malfunctions from loose coil contacts and broken gradient coils were identified [14]. In addition, it was observed that the individual magnets showed great differences in their drift of the B_0_ field with more than 90 Hz/day for a magnet of the first generation down to 0.5 Hz/day for the latest Terra magnet. However, even within the same magnet generation the difference in B_0_ drift was substantial with a range between 0.5 and 2.6 Hz/day for the Terra magnets within the network [15]. Unfortunately, GUFI’s published QA data is limited to only four participating sites, as the focus shifted towards the two travelling heads studies performed by the network [16, 17].

The first traveling heads study was conducted with UHF systems of the first and second magnet generation [16]. Within one year the same two male subjects were scanned at eight sites using a protocol with typical 7 T sequences for high-resolution structural imaging. Since the subjects were MR scientists, they could operate the MR systems themselves, which reduced the influence from having different operators between measurements. Intra-site variability was also assessed by repeating the scans at three sites.

Similar to the phantom QA, largest differences were found during adjustment scans in B_1_^+^ maps between the two variants of the RF coils. The transmitter calibration method provided by the MR vendor yielded a 14% higher transmitter reference voltage for the two 24-channel receive head coils compared to the 32-channel receive head coils. A dedicated B_1_^+^ mapping scan with the 3D DREAM method [18] revealed even a 26% difference. Details of the RF coil’s hardware components are proprietary information not accessible to the customers. However, it was expected that differences between the 24- and 32-channel head coils were not only regarding higher receive channel count but also regarding the transmitting birdcage coil. This assumption was underlined when one of the 24-channel coils was replaced at one site between two scan sessions and both datasets yielded consistent results.

Furthermore, an incorrect power calibration was identified in the turbo spin echo protocol at one site. This led to reduced flip angles and an elevated prediction of specific absorption rate (SAR) in comparison to the other sites. Consequently, the vendor was required to recalibrate the system in a service visit. After a new tune up of the RF system the transmitter efficiency and linear range were matched with those of the other systems. This incident highlighted the importance and benefits of networking and multi-center studies.

**Fig. 1:**
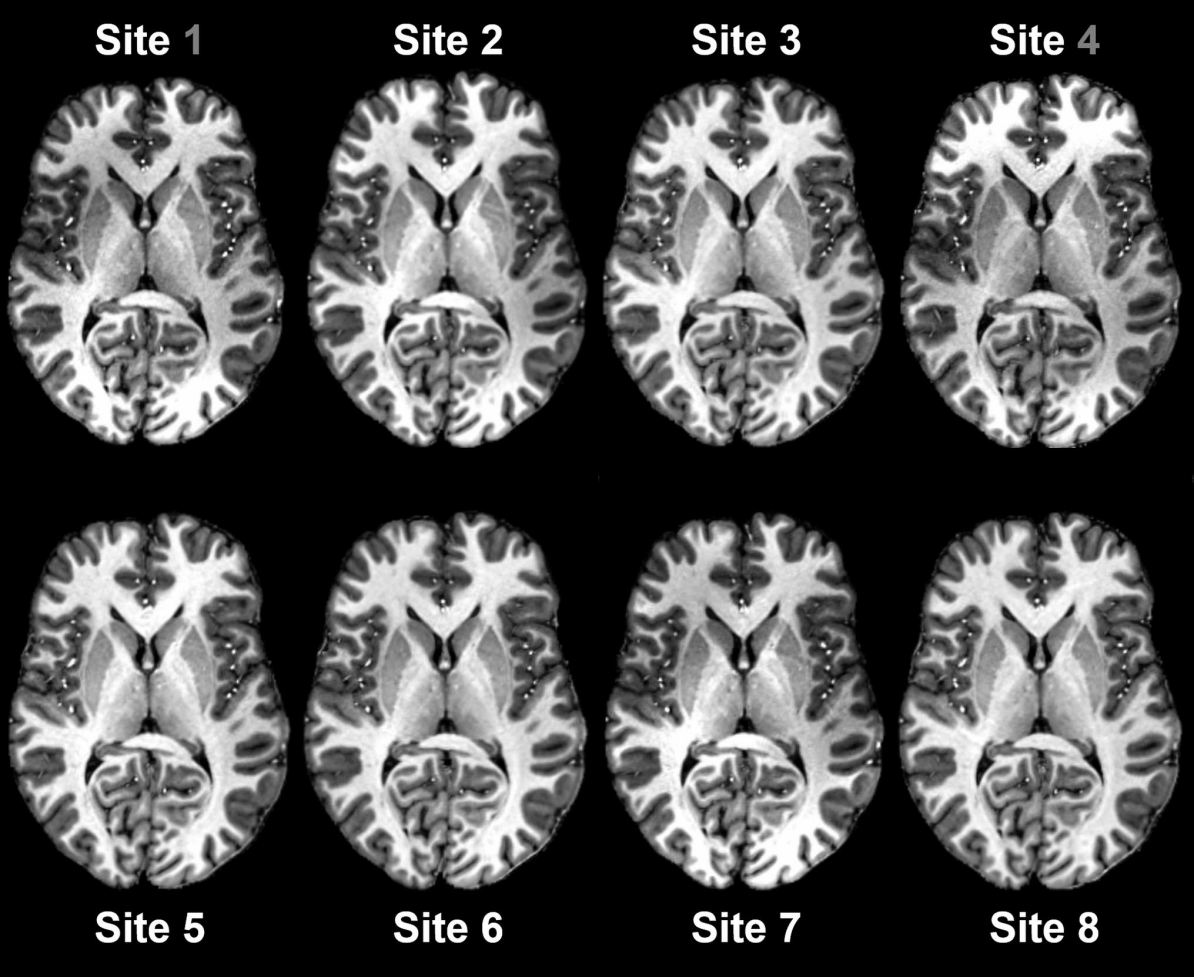
MP2RAGE uniform images from the GUFI travelling heads study [16]. Horizontal slice through the brain of one subject measured at different 7 T sites. 24-channel coil sites (1 and 4) are indicated with a dark grey numeral. Data were co-registered and brain extracted. MP2RAGE images show high agreement in image contrast at different sites. Reproduced with permission from Springer Nature

A key structural sequence at 7 T is the T1-weighted MP2RAGE [19] which can be found in almost all brain protocols from neurocognitive research studies to clinical scans (Fig. 1). A volumetric analysis on these images revealed small inter-site differences of 5% and a very high intra-site reproducibility with 0.6% difference. With the exception of the cerebellum and brain stem where the transmit field of the RF coils dropped notably, the coefficient of variation (COV) for T1 relaxation times in brain structures was found below 1%. For time-of-flight angiography, a high agreement was found in measured arterial contrasts with 3–4% difference in the intra- and inter-site comparison, respectively, while veins showed only minimally larger deviations in susceptibility weighted imaging (SWI) [20]. Both sequence types are also of high importance for clinical imaging at UHF.

**Fig. 2:**
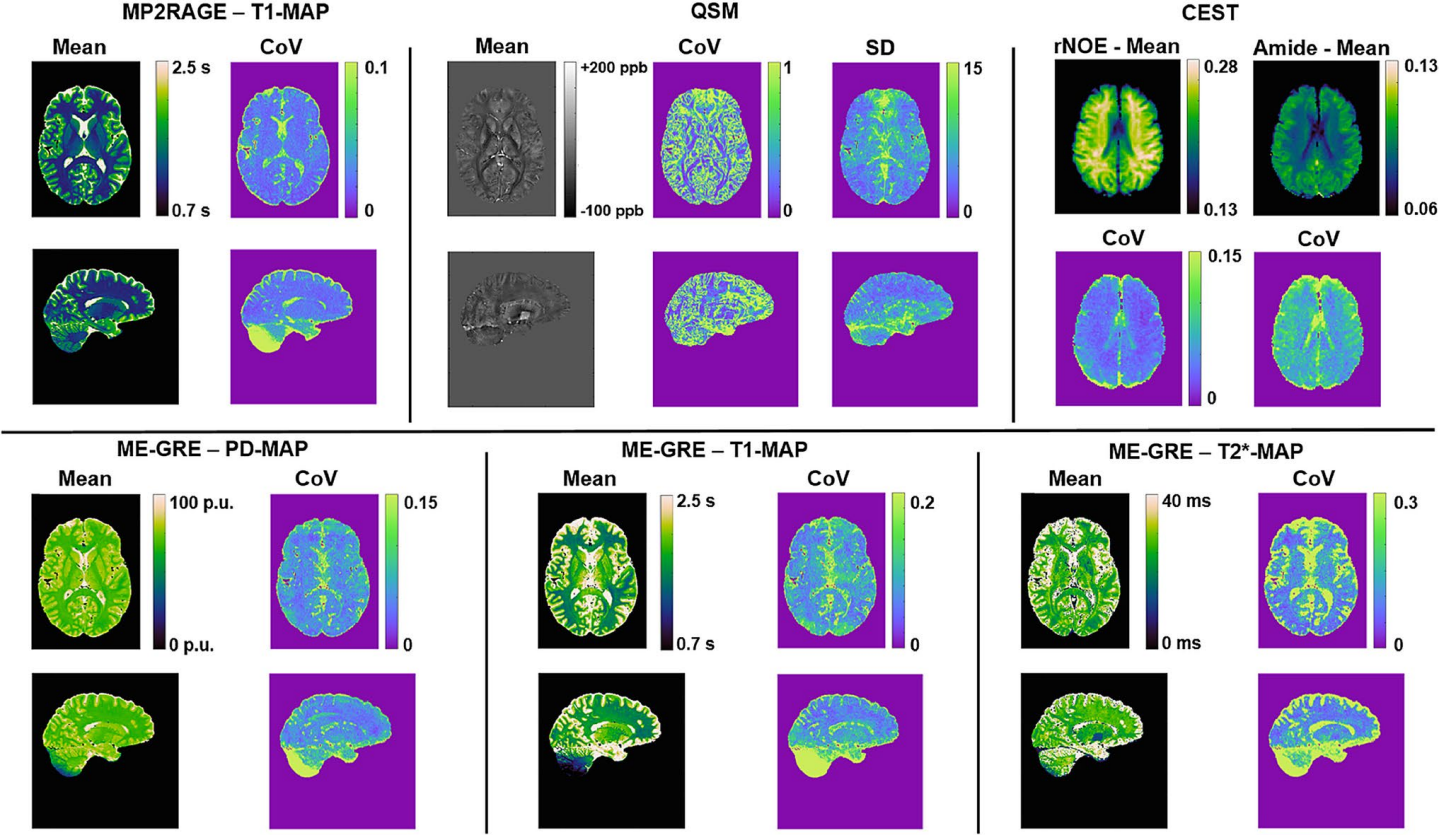
Reproducibility of quantitative imaging methods from GUFI’s second travelling heads study [17]. The mean and the CoV for all examined imaging methods are shown for the dataset of one subject. Note that the scale of the CoV maps has been adjusted to provide similar image contrast between methods. The T1 mapping with the MP2RAGE approach proved highly reproducible between different sites. The reproducibility drops for inferior regions like the cerebellum, where B_1_^+^ is low, especially for the C1 sites with the 24-channel coils. For the QSM maps, the standard deviation between sites is also shown to visualize more difficult regions independent of the normalization to the ppb scale. QSM had higher deviations near cavities (e.g., frontal hot spot near paranasal and frontal sinuses) and CSF-tissue boundaries. CEST rNOE had good agreement between sites and no hot spots showed up in the examined slab except for tissue/brain boundaries. The relaxometry with the ME-GRE approach showed lower reproducibility in regions with low B_1_^+^, e.g., cerebellum and brain stem analogous to the MP2RAGE, but also a gradient between the center and peripheral regions was found especially for the T1 maps. The higher central variability was driven by the differences between the measurements with different RF coils where input power was limited/different [17]

Around 3 years after the first multi-center study the same subjects visited again ten 7 T sites of the GUFI network, now including also systems of type Magnetom Terra [17]. This time, state-of-the-art quantitative imaging methods were addressed by evaluating the reproducibility of quantitative susceptibility mapping (QSM) [21], chemical exchange saturation transfer (CEST) [22], and multi-parametric T1/T2*/PD mapping [23]. In addition, brain volumetry and T1 mapping as performed in the first study was repeated with an updated MP2RAGE protocol utilizing now an HS4 inversion pulse instead of TR-FOCI, an improved B_1_^+^ correction, and image processing pipeline. The reproducibility stayed at similarly high levels as in the previous study and was found highest among all quantitative methods applied (Fig. 2) [17]. QSM yielded most reproducible results for the dentate nucleus, where the COV ranged between 2 and 4%, whereas higher variations were identified near air cavities and vessels with a maximum COV of 13%. Interestingly, the study again identified a substantive transmitter performance difference among the sites. The acquired relayed nuclear Overhauser effect (rNOE) maps of the CEST data showed a bias between the Terra sites and those of older magnet generation with a nearly 10% higher rNOE contrast in gray and white matter measured at the Terra sites [17]. A thorough investigation revealed that the long pre-saturation phase of the CEST sequence caused high thermal stress of the RF power amplifier (RFPA) leading to an altered effective pre-saturation RF amplitude between the sites with individual age or type of the RFPA. A calibration method was applied with additional scans conducted with the GUFI QA phantom available at all sites, which rendered a correction factor that could be applied to the rNOE maps. Repetitions of the calibration procedure at single sites were highly reproducible assuming a constant systematic error. After correction, the bias was reduced to 1.4% in gray and white matter tissues between the scanner generations. On the other hand, for the Amide peak and magnetization transfer (MT) contrasts either 7% smaller (Amide) or higher (MT) signal was measured at the Terra sites compared to the other systems [17]. The reasons for this systematic difference could not be explained but provide motivation for a comprehensive and well-considered QA for such multi-center studies with highly complex and novel data acquisition methods. The PD maps obtained with the multi-parametric mapping approach yielded nearly as reproducible inter-site results as the MP2RAGE T1 maps. For T1 and T2* relaxometry higher variations up to 9% (T1) and 12% (T2*), respectively, were found. However, due to software differences, this technique could not be implemented at the Terra sites at the time of the study [17]. A few years later, the repeatability of multi-parametric mapping was tested in a different travelling head study across five Terra sites [24], yielding generally good and consistent results, but the authors also observed large COV of around 50% for MT in the temporal lobe, indicating low repeatability.

### UK7T

**Fig. 3:**
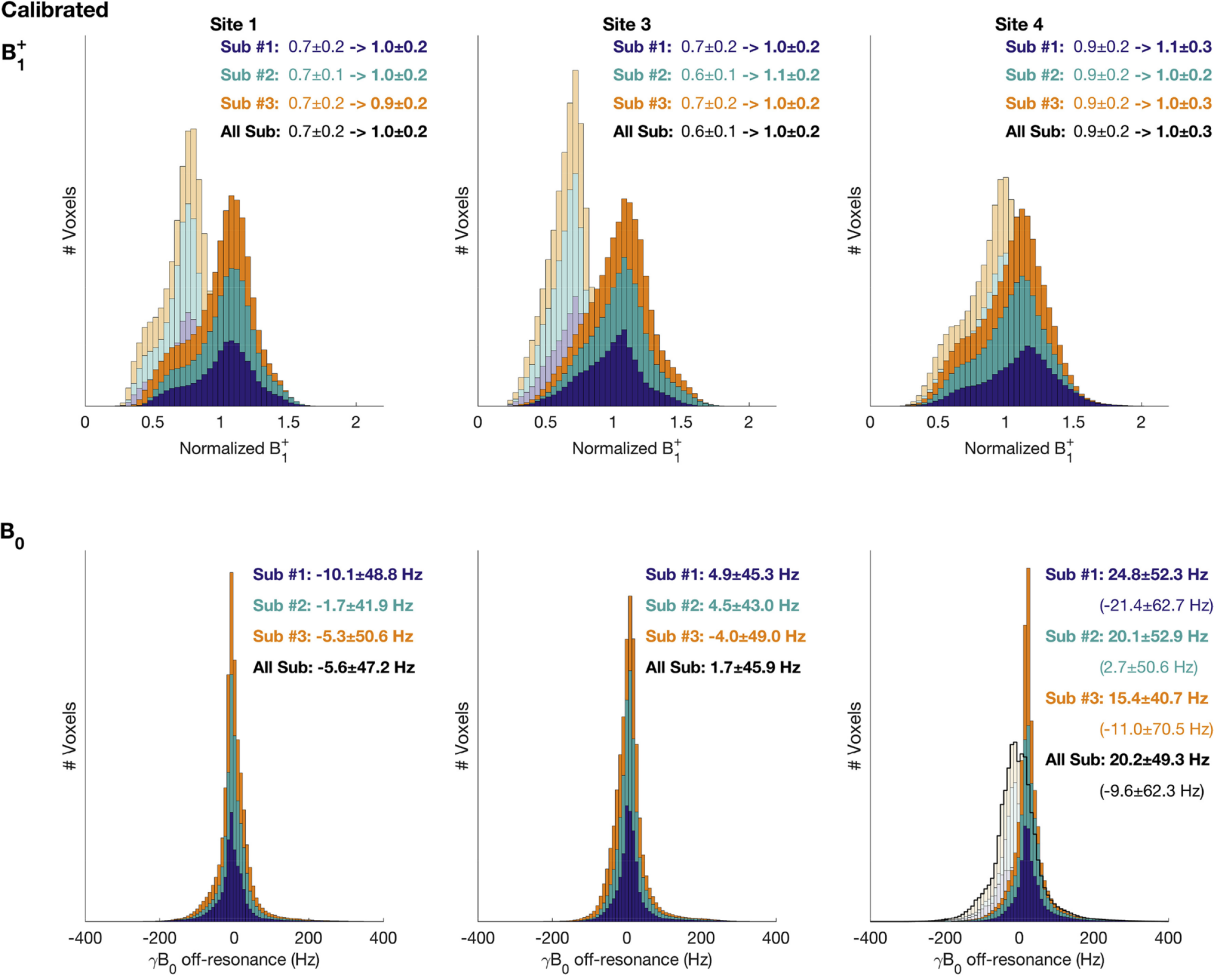
UK7T study on multi-site harmonization of neuroimaging protocols [25]. Whole-brain voxel-wise distributions of scanner calibration (B_1_^+^ and B_0_) measured on three subjects. Top: The effect of manual calibration of transmitter gain. The faint histogram shows the results of the vendor’s own calibration. The solid histogram shows the distribution after calibration using DREAM flip-angle maps. Bottom: B_0_ distributions. At two sites the distribution after the vendors automatic calibration is noticeably broader than the other scanner models (faint and black outline). A satisfactory shimming result is seen after manual shimming of the linear z gradient (solid histogram) [25]

Another UHF network was established in the UK with funding from the Medical Research Council between 2016 and 2019 [13]. Five sites in the UK participated using three different 7 T scanner models, manufactured by two different vendors. The aim of the network was to establish a set of harmonized structural and functional neuroimaging sequences and protocols, and to demonstrate that UK7T can act as a platform for large-scale multi-site studies. In a similar way to GUFI, the focus also quickly shifted from distributed phantoms and QA measurements towards implementing their own traveling head studies [25, 26]. To test the level of cross-vendor harmonization of the sequences the same subject was measured at all five sites, and additionally four times at one reference site [25]. In line with the observations from the GUFI network, Clarke et al. reported that B_1_^+^ calibration was essential for this study [27]. A greater difference between models and a greater underestimation of the required calibration compared to manual calibration was found in the automatic adjustments of the manufacturers. Figure 3 shows the effect of manual calibration of transmitter gain as well as on B_0_ shimming. The study also highlighted differences in gradient non-linearity correction between vendors leading to a 40-times greater variance in the quantitative assessment of cortical thickness derived from MP2RAGE data across sites than for intra-site comparisons. However, the difference could be corrected retrospectively.

A follow-up study on QSM and T2* imaging was conducted on two healthy volunteers from each of the five sites, i.e., ten in total, where each subject was scanned five times at their home site, and once at the other sites of the network [26]. Again, differences in geometric distortion across scanners were observed and the authors recommended to use a non-linear registration method instead of rigid-body registration to reduce the inter-scanner variability of cortical QSM. By comparing the reported COV values for reproducibility of single echo QSM data between the two travelling heads studies from UK7T and GUFI, a very high agreement was found for example in the basal ganglia structures substantia nigra (6.1% vs. 5.8%), red nucleus (8.8% vs. 5.6%), caudate nuclei (7.6% vs. 7.8%), putamen (5.2% vs. 6.7%), and globus pallidus (5.5% vs. 6.7%) [17, 26].

### Special hardware QA at UHF

#### RFPA

**Fig. 4:**
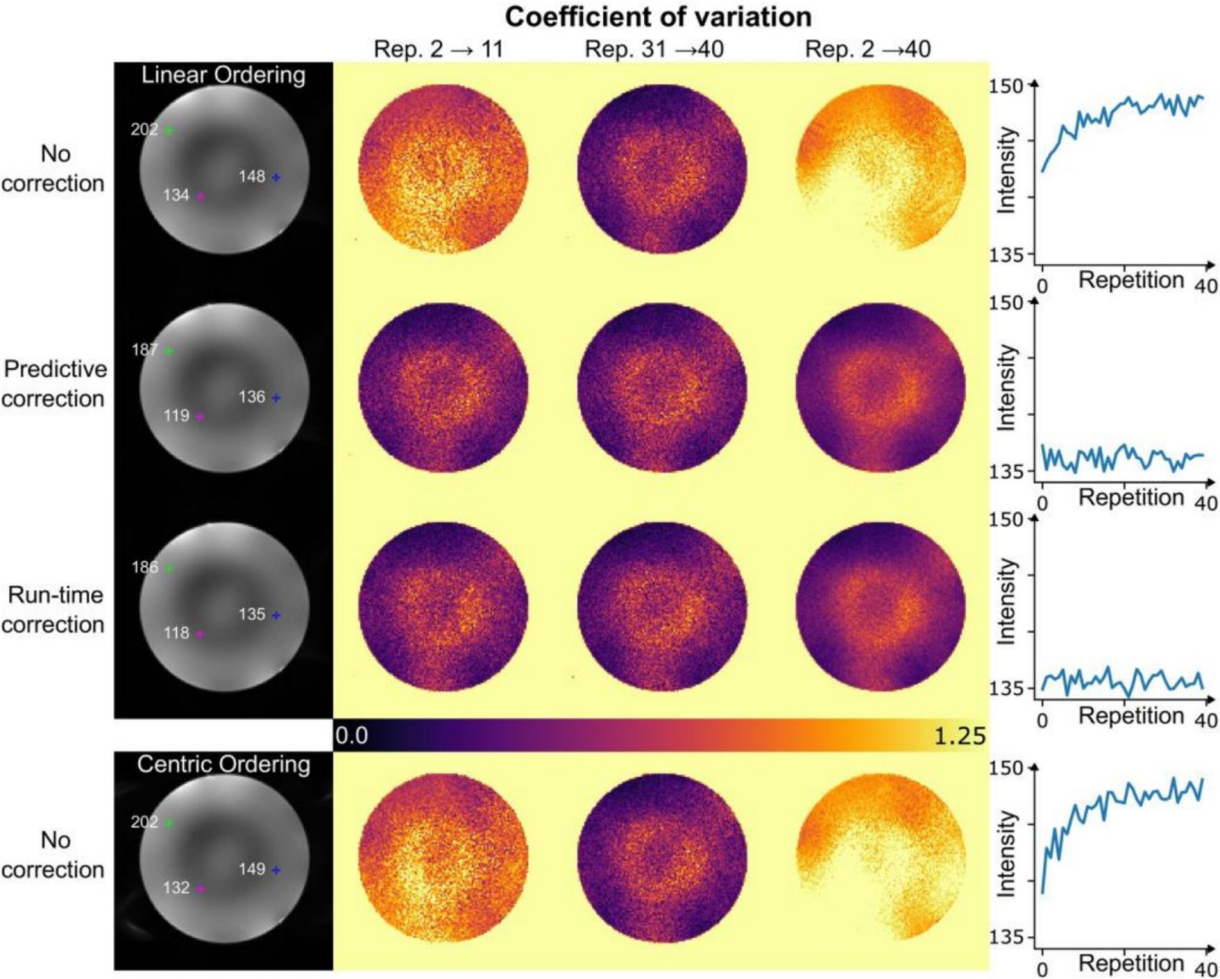
A comparison between no RFPA drift correction, predictive RFPA drift correction, and run‐time RFPA drift correction in a phantom study [29]. The initial repetition is excluded from the analysis to mitigate the impact of the transient state. The first column displays reconstructed images from the 40th repetition. The intensities of three selected voxels are depicted, revealing higher intensity in the absence of correction. COV is computed across three intervals: the first 10 repetitions, the last 10 repetitions, and all repetitions combined. The application of RFPA correction results in more consistent COV values. The last column showcases the evolution of the voxel marked with a blue cross across all repetitions. Notably, both predictive and run‐time corrections yield more stable intensity profiles in comparison to the scan without any correction. The final row presents the same scan with a centric ordering scheme, illustrating the greater sensitivity of centric ordering to RFPA drift [29]

The success of the two major networks GUFI and UK7T, as well as their cooperation and regular exchange at scientific meetings led to further consortia, which addressed dedicated QA measurements at UHF. A drift in the transmitter chain was already identified in the travelling heads study with the single channel transmit head coil [17]. At UHF, such drifts become more problematic in pTx scans, where multiple RFPA outputs are utilized to drive a pTx RF array coil with 8 or even 16 transmit channels [28]. Therefore, Aghaeifar et al. performed a dedicated RFPA drift assessment at five sites comprising MR systems between 7 T and 11.7 T [29]. While all RFPAs were manufactured by the same vendor (Comet XYLON International GmbH, Germany), some systems used 1 kW RFPA units and others were fitted with 2 kW RFPA units. Inter- and intra-pulse drifts were investigated by employing a sequence with only RF pulses but no gradients, and by analyzing the detected forward RF power at the directional couplers of each RF transmission line. The authors found a three-fold difference in magnitude drift within RFPA units of former and more recent design at a single site, but also large differences between sites with average drift values of 7.9% for a 7 T Plus, 10.9% for a 7 T Terra, up to 25.6% for a 9.4 T, and 15.7% for a 11.7 T MR system [29]. In case the RFPA drift remains unaccounted, it could lead to an over- or underestimation in the supervision of magnitude SAR and hence, result in result in interrupted scans and an unreliable protocol for clinical scans. Furthermore, Aghaeifar et al. also demonstrated an influence on B_1_^+^mapping techniques and, consequently, on quantitative techniques like T_1_ relaxometry, which use the B_1_^+^ map to correct for residual flip angle inhomogeneities [29]. These effects, however, can be mitigated with predictive and run-time drift correction as shown in Fig. 4.

#### Gradient field interactions

**Fig. 5:**
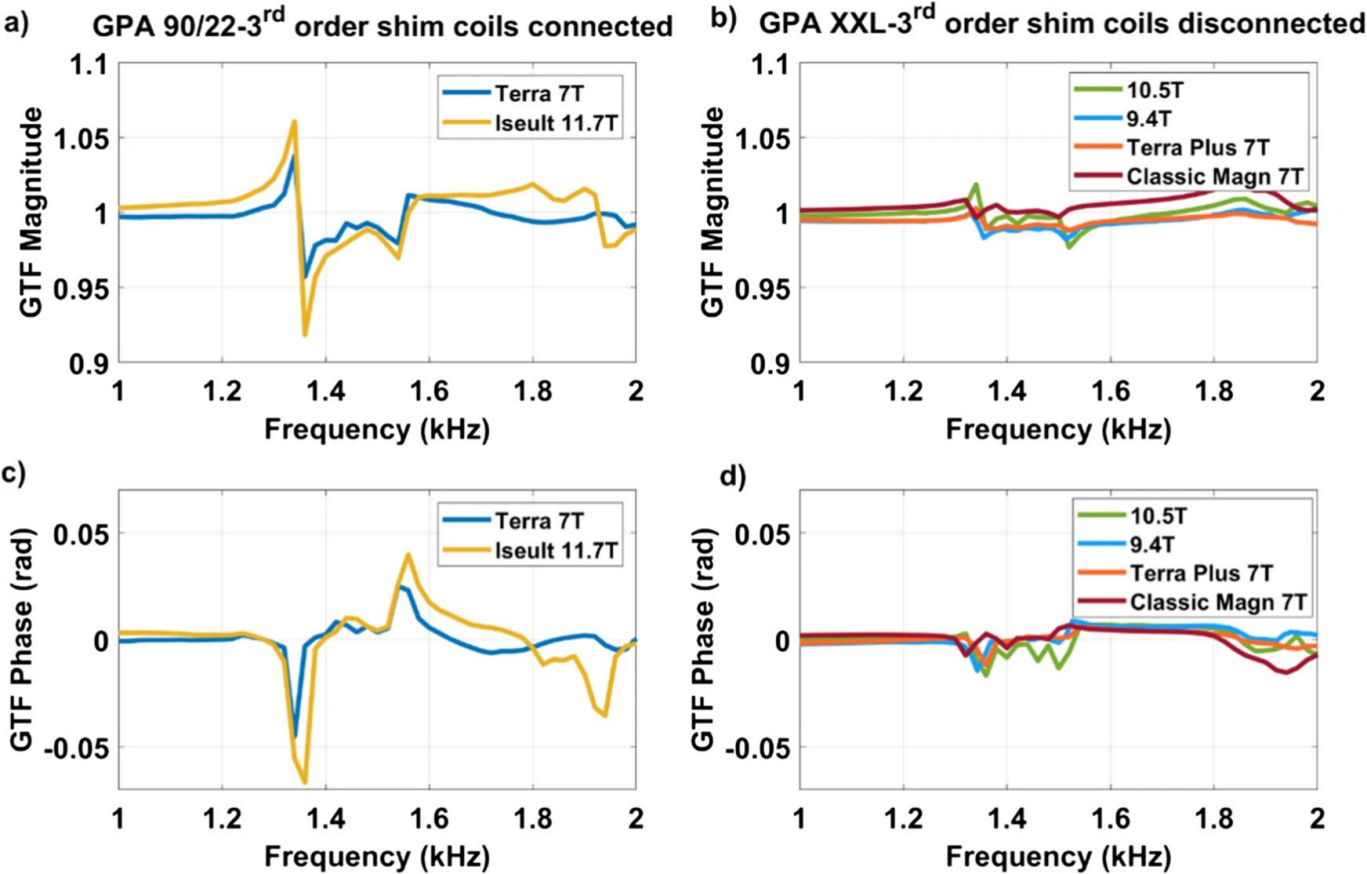
On the possible influence of third-order shim coils on gradient–magnet interactions from Boulant et al. [30]. Self-term Z GTF measurements for the SC72 whole-body gradient coils when driven by the GPA 90/22 (third-order shim connected) versus GPA XXL (third-order shim disconnected) at various field strengths. **a**, **b** Magnitude and c, d phase in the GTFs are provided [30]

Another multi-center study performed at sites with MR systems operating between 7 and 11.7 T investigated the coupling of the same whole-body gradient coil with the third-order shim coil [30]. Boulant et al. found great differences depending whether the third-order shim coil was connected or not. Gradient transfer functions (GTF) were analyzed from three 7 T systems, as well as from single 9.4, 10.5, and 11.7 T systems. Despite the lower static magnetic field, a higher peak at 1350 Hz was found at 7 T with connected third-order shim compared to systems of higher magnetic fields with third-order shims disconnected at the filter plate (Fig. 5). Although the authors could not fully explain how the vibrations couple to the shim coils and how it exactly influences the gradient field, due to lack of proprietary information from the MR vendor, the study addressed its influence on inter-site variability on echo-planar imaging but also on structural imaging that utilizes gradient spoilers as in MPRAGE [30].

#### RF coils

Currently, only two commercially available UHF MRI scanners have FDA clearance: the Siemens Magnetom Terra (approved in 2017) and GE’s Signa 7.0 T (approved in 2020) [2, 3]. The standard coil at the Siemens system is a 1Tx32Rx head coil, while GE uses a 2Tx32Rx brain coil. However, Siemens’ recent upgrade to the 8-channel pTx Terra.X system marks the shift towards advanced pTx technologies, which will likely become the focus of future UHF coil QA efforts. A significant challenge in UHF imaging is the limited availability of commercially produced UHF coils, especially full-body coils, which are currently unavailable. As a result, researchers and clinicians must primarily rely on custom-built local transmit/receive coils to extend the FOV [31–33]. The absence of standardized, full-body UHF coils highlights the growing need for commercially available solutions to expand clinical and research applications.

A dedicated QA for UHF coils is essential for maintaining imaging performance across multiple centers. Although RF coil comparisons of different UHF coils exist [34–36], dedicated multicenter QA studies of one type of UHF coil have not been published so far. In the context of RF coils with multiple transmit channels, there is an increasing need to monitor the performance of the RF coils over time [37]. Typically, this kind of hardware is produced in small numbers either by coil vendors or by some UHF sites themselves, and, thus are more handcrafted prototypes than robust series productions.

**Fig. 6:**
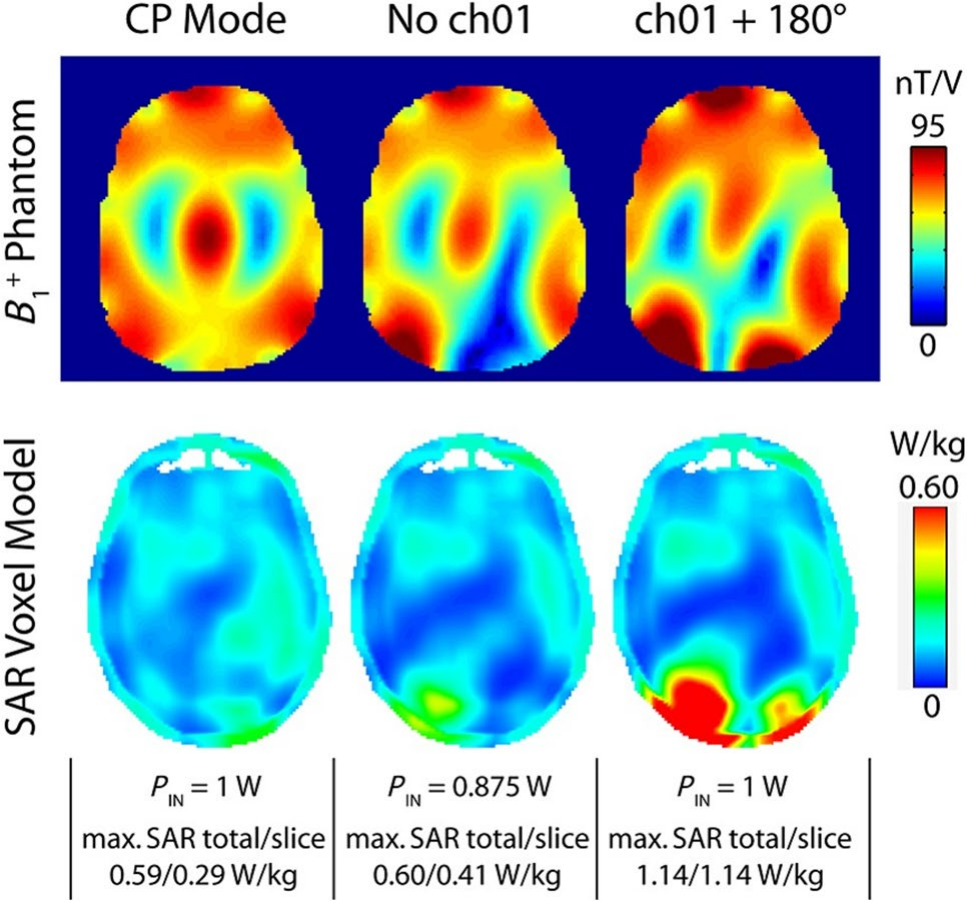
Impact of element failure in a custom-built eight-channel RF array on B_1_^+^ in a phantom (top row) and SAR in the Duke model (bottom row) [39]. As E-fields in the CP mode (left column) tend to interfere destructively between neighbouring elements, the absence of the posterior transmit element locally increases SAR (middle column). A phase shift of 180° causes twice the maximum local SAR across the model (right column). In the absence of hardware supervision, such malfunctions can be detected via rapid B_1_^+^ mapping prior to in vivo scanning (top row). Reproduced with permission from John Wiley and Sons [39]

Furthermore, RF coil QA is important for ensuring safety at UHF. Best practices for safety testing of experimental RF hardware have been recently published by a working group of the International Society of Magnetic Resonance in Medicine (ISMRM) [38]. Transmit and receive functionalities of the RF coil can be verified using predefined tests. As malfunction in a Tx element will probably change the RF field (Fig. 6), Hoffmann et al. recommend to compare scout images or rapid B_1_^+^ maps with reference images or maps before each in vivo scan [39]. In addition, at regular intervals RF coil QA tests should include measurements of the scattering parameters on the bench, and verifying the power linearity of the RF coil to detect coil degeneration or failure. To guarantee stability of transmit and receive coil paths on a long-term basis noise and SNR on each channel should be monitored as well [37]. To expedite the transition of custom coil systems from prototypes to clinical use, a restricted SAR protocol has been established [40]. Users of custom-built hardware need to know the reliability and failure tolerances of all RF components to assess the impact on their measurement results [41]. SNR is a critical factor in QA comparisons; however, SNR can be influenced by various system variables such as resonance frequency, flip angle accuracy, transmitter gain, coil loading, and scan parameters. Factors like scan acceleration (e.g., parallel imaging), image reconstruction, and postprocessing also impact SNR [42]. Due to these influences, B_1_^+^ field homogeneity and efficiency are often considered more reliable parameters for comparing the performance of different transmit coils [36]. However, precise B_1_^+^ mapping remains a challenge at UHF. Several new B_1_^+^ mapping techniques have been introduced in the past, but to date none has emerged as a standard [43].

RF non-uniformity in both transmit and receive coils has long been recognized as a significant issue in multicenter UHF studies [44]. Various antenna types are commonly used for pTx applications, including conventional segmented loops [45], microstrip transmission line elements [46], and dipole antennas [47], as well as combinations of loops and dipoles [48]. Despite these developments, there remains a lack of comprehensive multi-site comparisons of different antenna and coil geometries, including those already available commercially. The future of UHF coil QA will increasingly focus on pTx technologies. However, care must be taken to characterize and track the image quality of such systems and detect possible errors to ensure long-term stability [9, 49]. As UHF systems evolve, standardized QA protocols and phantoms will be critical to ensuring consistency in coil performance and safety across different imaging centers.

### Phantoms for QA

Choosing the right phantom for QA measurements is a key requirement to obtain reliable results. However, especially with increasing magnetic field strength, this is not an easy task. For 1.5 and 3 T systems standardized phantoms were described in the published literature by large networks. In 2006, Friedman and Glover published their experience in using the Functional Bioinformatics Research Network (fBIRN) phantom over several years for regular QA testing [50]. The phantom was made of doped agar gel filled in a spherical plastic vessel to approximate T1 and conductivity of brain tissue at 3 T, and to match the needs for stability tests in functional MRI (fMRI). A gel is typically preferred to fluids as it avoids a long settling time after movement to the magnet’s isocentres and ensures less influence from vibration. Latter is of paramount importance for protocols that stress the system with high-duty-cycle EPI imaging to capture signal drift or fluctuations. Furthermore, for safety testing of implants and RF coils, a gel is often used as it reduces thermal convection [51]. The fBIRN phantom and protocol have been extensively used over the years, both at 3 T and at 7 T. Its stability measurements were also part of the GUFI QA measurements performed at 7 T [14].

**Fig. 7:**
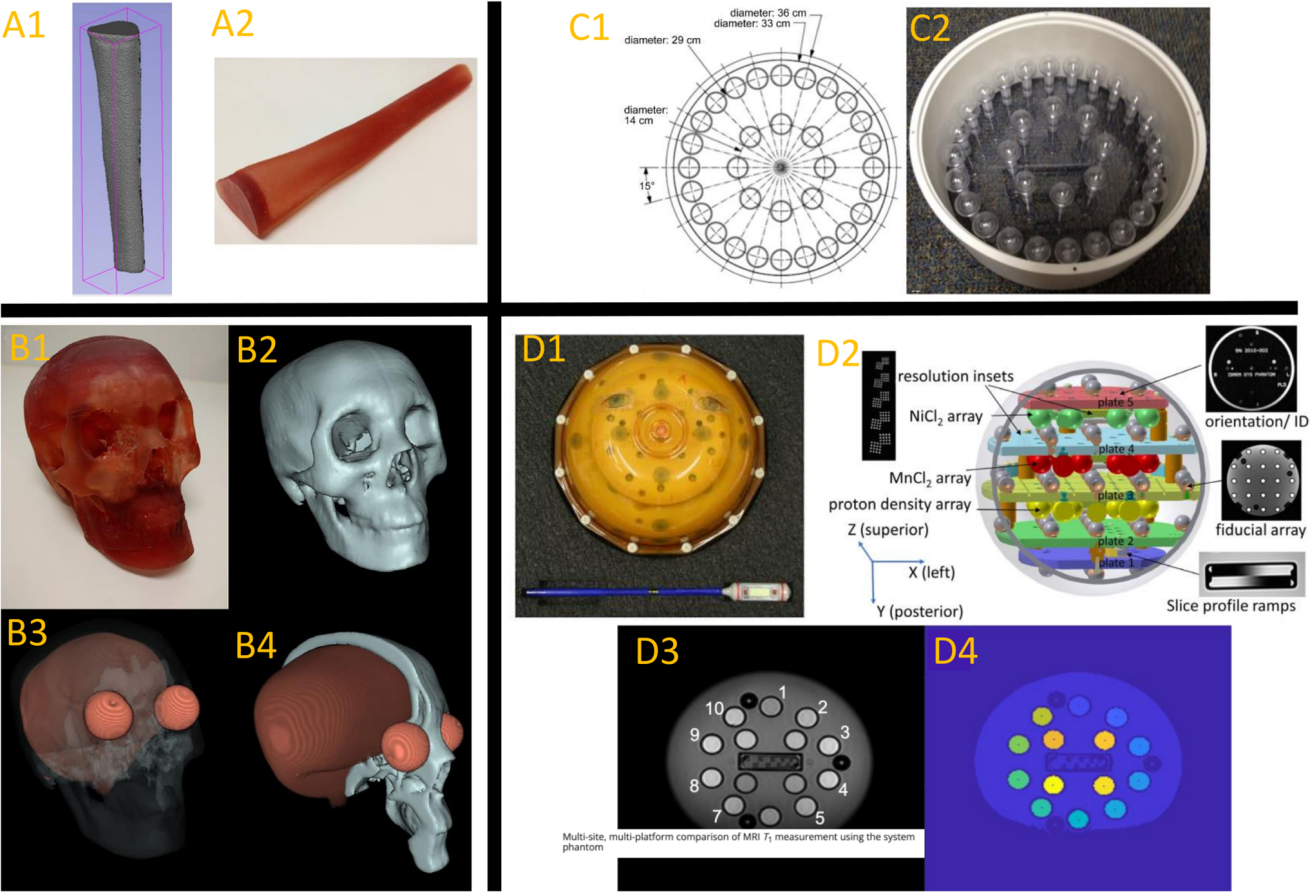
Example of different phantoms. On the left side anatomical structure mimicking phantoms are shown in **A** and **B**. Manual contour of a cortical bone (A1) STL model and the 3D printed cortical bone model in resin (A2) are given. Reproduced with permission from John Wiley and Sons [76]. In (B1) a 3D printed skull in photopolymer resin, and in (B2) a volume-rendered image of skull with realistic bony details with (B3) surface rendering showing skull, brain, and eyes with variation in signal corresponding to variation in colors. (B4) cross-section of complete skull phantom demonstrates air cavities, balloon mimicking brain tissue, and eyes in the orbital cavity. Reproduced with permission from John Wiley and Sons [76]. A diagram (C1) and an image (C2) of the Quantitative Imaging Biomarkers Alliance dynamic contrast-enhanced (QIBA DCE)-MRI phantom. Reproduced with permission from John Wiley and Sons [42]. In (D) an example coronal slice of the ISMRM/NIST system phantom through the NiCl2 array and resulting segmentation are shown [76]. (D1) Top view of phantom showing eye decals. (D2) Schematic of system phantom showing different structures, arrays, resolutions and slice profile insets [77]. (D3) exemplarily shows a short inversion time image used for identification of sample spheres and (D4) shows the segmentation with sample sphere centers identified [78]

The Alzheimer’s Disease Neuroimaging Initiative (ADNI) phantom is another example of a widely used phantom for a multisite study that is collecting data from 1.5 to 3 T systems since 2004 [52]. The ADNI phantom consists of spherical inclusions inside a water-filled clear urethane shell. Spherical inclusions made of copper sulfate filled polycarbonate are used to assess geometrical measurements for linear scaling and to determine gradient nonlinearities, as well as for SNR and CNR measurements for small shells of varying T1 relaxation times. In 2012, the National Institute of Standards and Technology (NIST) collaborated with the ISMRM ad-hoc committee on Standards for Quantitative MRI (SQMR) to develop a system phantom for quantitative assessment of T1, T2, proton density, and geometric distortion [53]. The NIST/ISMRM system phantom (Fig. 7) was developed with experiences obtained from the ADNI phantom described before and one phantom proposed by the American college of Radiology (ACR) [54], which also used resolution insets, slice profile wedges, and grids to determine geometric distortion and contrast.

It consists of multiple layers of sphere arrays with known T1, T2, and PD values, but more importantly, this system phantom has been commercialized to be available to all MR customers as a precision machined system phantom [55]. So far, reference relaxation times are available from the NIST for 1.5 and 3 T, only, but two studies recently published T1 values of the 14 T1 spheres in the NIST/ISMRM system phantom measured at 7 T [56, 57]. However, the authors of both studies had to modify the housing of the phantom to fit into the 7 T RF coils used indicating that its current form is not suited to become a system phantom for 7 T. In addition, strong limitations from B_1_^+^ inhomogeneities were reported with a span between the highest and the lowest B_1_^+^ in the spheres of 30%, when the spheres were measured in air without outer housing [57], and up to 40% in the case of a modified phantom housing [56].

On one hand, a large fluid-filled vessel is needed for susceptibility matching. This is particularly important for UHF as susceptibility artifacts become more pronounced with increasing static magnetic fields [58]. On the other hand, water, which is typically used to avoid such B_0_ variations between samples in a phantom at 1.5 and 3 T, has a relatively high relative permittivity of around 80. The much larger permittivity of the phantom compared to the surrounding air leads to B_1_^+^ interference patterns and notable FA variations even across a head-sized phantom [59, 60]. Instead of water, vegetable or mineral oils are often used as oil produces a very uniform B_1_^+^ distribution. Due to the offset in resonance frequency between oil and water it may introduce chemical shift artifacts, which can also influence a quantitative QA measurement depending on the bandwidth and RF pulse sequence.

Besides the influence of the relative permittivity on the B_1_^+^ distribution the phantom filling at UHF also needs to take the conductivity into account. Due to the increased load-dependency of UHF RF coils in general, and in particular for multi-channel pTx coils, where load-dependent inter-element coupling needs to be considered, too, low-conductive oil could lead to misleading results. Salt is typically incorporated as an additive to control for electrical conductivity in aqueous phantom fillings, and hence, allows for realistic loading conditions of the RF coil. For some applications, a phantom that loads the RF coil well and even leads to a notable inhomogeneous B_1_^+^ may be desirable for assessing the reproducibility of pTx techniques.

For example, Gras et al. presented a concept for calibration-free pTx based on universal pulses (UP) [61]. The authors demonstrated the efficiency of robust plug and play RF pulses for brain imaging at 7 T with respect to head size, anatomy and position variations, and hence, with no need for lengthy subject-specific preparations [62]. The feasibility of UP for multi-center studies has been presented by Wu et al. for healthy volunteers [63]. Currently, the SCAIFIELD consortium [64] investigates the visualization of structural brain alterations in the cerebellum and brainstem in patients with spinocerebellar ataxias with a 7 T pTx protocol that uses UP. A derivative of the GUFI phantom and a QA protocol that quantifies the reproducibility of phantom-specific UP excitation will be used by the consortium [65].

A higher reproducibility and easier replacement of the phantom is ensured by printing the housing in nylon instead of gluing acrylic glass compartments as in the previous GUFI version. The phantom is made of three compartments with varying dielectric properties, i.e., brain, muscle, and lipid [65]. In general, different media have been investigated as phantom filling for UHF, such as oil [66], sugar [67, 68], alcohol [69], and PVP [14, 70, 71]. Tissue simulating liquids made of sugar, salt, and deionized water were used mainly for coil validation purposes as the ingredients are easily available from supermarkets, straight-forward to prepare, and allows to define the permittivity and conductivity. Its applicability for imaging purposes, however, is limited due to low SNR and a sedimentation of sugar over the time.

Isopropyl alcohol, for example, introduces difficulties in the housing material as it quickly leads to microfractures in acrylic glass and other plastic materials. PVP-based phantoms emerged as a promising alternative as they are also easy to prepare, non-toxic, higher in SNR than sucrose-based phantoms, and allow to simulate both, dielectrical properties as well as favorable relaxation times for a variety of applications [70]. On the other hand, it has been reported that for high PVP concentrations, the phantom becomes prone to degradation. Differences in electrical properties have been observed over 1 year of usage [70].

Depending on the specific application, phantoms may be added with metabolites for MR spectroscopy [65, 72], or with vials of varying iron concentrations for QSM [73], for example. The latter has been successfully deployed in a multi-center QA to evaluate the capability of QSM at magnetic field strengths of 3, 7, and 9.4 T [73]. Finally, another important aspect of phantom design is to include anatomical details (Fig. 7), either by utilizing a few different compartments [65] up to anthropomorphic phantoms [74, 75]. Brink et al. reported on the importance of including a spherical air cavity in their simplified head-size phantom as an important contributor to the B_1_^+^ asymmetries observed in vivo, and hence, for the RF coil validation purpose [71].

## Conclusion

This review highlights the critical role of multi-center QA in ensuring consistent and reliable data acquisition across UHF-MRI systems, especially with the growing number of 7 T installations. Networks such as GUFI and UK7T have demonstrated the ability to monitor reproducibility and system performance effectively. Multi-center QA studies, particularly those involving in in vivo travelling head assessments, have shown high reproducibility across MR systems of different generations and vendors. However, advanced QA measurements that apply high RF or gradient duty cycles have revealed significant drifts, affecting imaging data. The lack of standardized phantoms and UHF coils further underscores the need for a dedicated QA protocol, which is essential for identifying system variations, addressing hardware inconsistencies, and standardizing imaging procedures. As UHF MRI becomes more prevalent in research and clinical applications, robust QA protocols will be vital for ensuring data harmonization and supporting large-scale, multi-site studies.
